# Dermoscopy of late-onset nevus comedonicus^⋆^^⋆⋆^

**DOI:** 10.1016/j.abd.2020.03.018

**Published:** 2020-09-14

**Authors:** Cesare Massone, Sanja Javor, Simona Sola

**Affiliations:** a Dermatology Unit, Galliera Hospital, Genova, Italy; b Surgical Pathology, Galliera Hospital, Genova, Italy

Dear Editor,

Nevus comedonicus (NC) is a rare hamartoma of the pilosebaceous unit, a subtype of epidermal nevus first described by Kofmann in 1895.^1^ NC manifests with linear or grouped papules and dilated follicular openings with keratotic plugs (resembling comedones) particularly on the face, trunk, and neck; in 50% of cases, it is present at birth, but can also develop during childhood (most commonly before the age of 10 years). The rare presence of skeletal and neurological abnormalities describes the nevus comedonicus syndrome.^2^ Histopathology shows keratin-filled invaginations of the epidermis, with absent or rudimentary sebaceous glands. Inflammation and subsequent dermal infiltrate have been described in some cases.^2^ Rarely, NC may present in adults;recently Zaniello et al., reporting an additional case of late-onset NC, reviewed the few cases described.^3^

A 72-year-old man with a previous history of stage IB cutaneous melanoma on the trunk in 2013 and prostate carcinoma presented in June 2019 with a keratotic plaque (2 × 1 cm in diameter) and a small comedo-like nodule (almost 1 cm in diameter) on the left calf, following a Blaschko line (Fig. 1). The lesions had appeared almost 25 years before, and the patient had been asymptomatic until the week before, when the lesions became very itchy. Dermoscopic examination showed a central keratotic plug surrounded by a white structureless area with scales and focal pale structureless red area without clear vessels (Fig. 2). The patient had been taking candesartan, hydrochlorothiazide, bicalutamide, simvastatine, and rabepreazole for years. General physical and neurological examinations were normal, and the patient denied a family history of analogous lesions. Histopathology demonstrated a cyst-dilated follicular opening filled with keratin, slight acanthosis of the epidermis, and hyperkeratosis with ortho- and parakeratosis. The follicular epithelial wall and the epidermis showed EHK with hypereosinophilic keratohyalin granules in the granular cell layer and perinuclear vacuolization (Fig. 3). Sebaceous and eccrine glands were not present. A focal discrete lymphohistiocytic infiltrate was present in the papillary dermis. Upon clinicopathologic correlation, the diagnosis of late-onset NC with EHK was made. The main differential diagnosis was inflammatory linear verrucous epidermal nevus (ILVEN), which typically presents in the first 6 months of life as a pruritic linear eruption on the lower limbs, arranged along the Blaschko lines. At histopathology, ILVEN shows psoriasiform epidermal hyperplasia with parakeratosis, alternating with orthokeratosis. Beneath the orthokeratosis, hypergranulosis is observed, while the parakeratosis overlies areas of agranulosis. Focal mild spongiosis with some exocytosis and even vesiculation may be present, together with a mild perivascular lymphocytic infiltrate in the upper dermis.^3^ The infiltrate observed in the present case was probably caused by irritation. EHK is characterized by compact hyperkeratosis with granular and vacuolar degeneration of the cells of the spinous and granular layers. It may be an incidental finding or may be observed in different settings, such as bullous ichthyosis, epidermal nevi variant, palmoplantar keratoderma variant, or disseminated epidermolytic acanthoma.^3^

**Figure 1:**
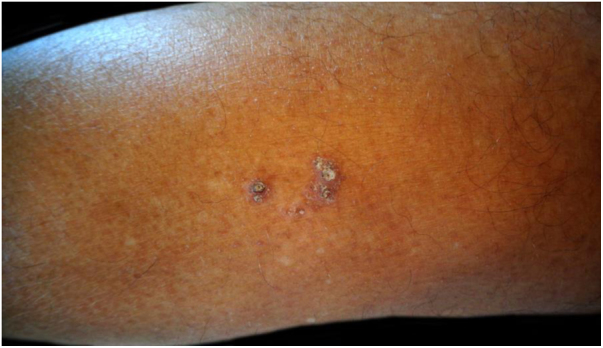
Keratotic plaque and small comedo-like nodule on the left calf following a Blaschko line.

**Figure 2:**
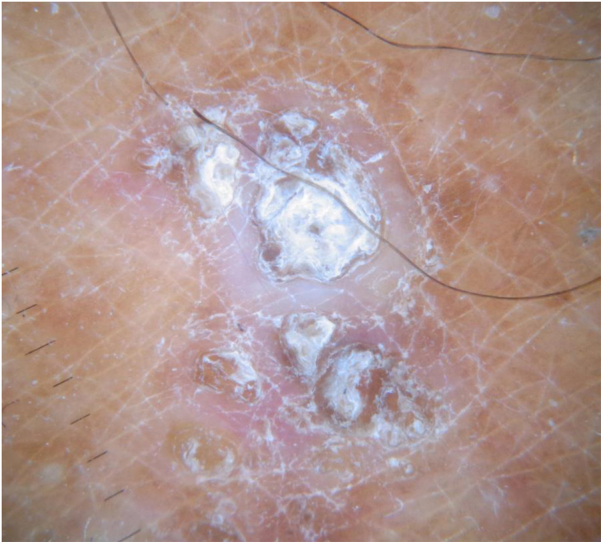
Dermoscopic examination showed a central keratotic plug surrounded by a white structureless area with scales and focal pale structureless red area without clear vessels.

**Figure 3:**
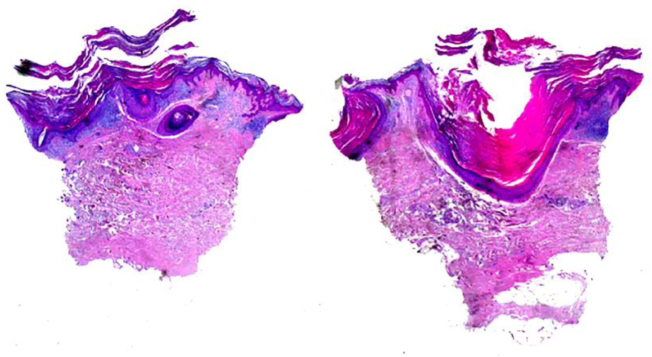
Cyst-dilated follicular opening filled with keratin, slight acanthosis of the epidermis, hyperkeratosis with ortho- and parakeratosis. The epithelial wall and the epidermis showed epidermolytic hyperkeratosis (Hematoxylin & eosin, ×20).

EHC in a NC has been reported in few cases in the literature reviewed by Zanniello et al.,^3^ who reported a peculiar case with late onset (55-year old woman). The present patient is an additional case of this rare histopathological variant of NC.

Dermoscopy of NC was reported in detail for only two young patients. Vora et al. described multiple, well-defined, structureless brown homogenous circular areas surrounding the keratin plugs.^4^ Kamińska-Winciorek et al. reported numerous circular and barrel-shaped, homogenous areas in light and dark-brown shades, with remarkable keratin plugs.^5^ In the present case, a central keratotic plug was observed, surrounded by a white structureless area with scales and focal pale structureless red area without clear vessels. The following dermoscopic differential diagnoses were considered for the present case: squamous cell carcinoma/keratoacanthoma, which shows an amorphous, yellow-white central mass of keratin, hairpin vessels, and/or serpentine vessels; common wart, which at dermoscopy presents multiple densely packed papillae, with a central red dot or loop, surrounded by a whitish halo; molluscum contagiosum, which displays a central pore in association with polylobular white-to-yellow amorphous structures, surrounded by blurred telangiectasia.

A topical treatment with methylprednisolone aceponate 0.1% for on week and urea 10% ointment twice a day as maintenance therapy was prescribed, with fast improvement of the pruritus and slight decrease of keratotic component.

## Financial support

None declared.

## Authors' contributions

Cesare Massone: Approval of the final version of the manuscript; design and planning of the study; drafting and editing of the manuscript; collection, analysis, and interpretation of data; intellectual participation in the propaedeutic and/or therapeutic conduct of the studied cases; critical review of the literature.

Sanja Javor: Approval of the final version of the manuscript; drafting and editing of the manuscript; collection, analysis, and interpretation of data; critical review of the literature; critical review of the manuscript.

Simona Sola: Approval of the final version of the manuscript; design and planning of the study; drafting and editing of the manuscript; collection, analysis, and interpretation of data; effective participation in research orientation; critical review of the literature; critical review of the manuscript.

## Conflicts of interest

None declared.
